# Effect of coffee agriculture management on the population structure of a forest dwelling rodent (*Heteromys desmarestianus goldmani*)

**DOI:** 10.1007/s10592-017-1016-9

**Published:** 2017-10-09

**Authors:** Beatriz Otero-Jiménez, John H. Vandermeer, Priscilla K. Tucker

**Affiliations:** 1 Department of Ecology and Evolutionary Biology, University of Michigan, 2019 Kraus Nat. Sci. Bldg., 830 North University, Ann Arbor, MI 48109-1048 USA; 2 Museum of Zoology, University of Michigan, 1109 Geddes Ave, Ann Arbor, MI 48109 USA

**Keywords:** Small mammal, Agriculture, Matrix, Landscape genetics

## Abstract

Most of the natural habitat in tropical regions exists as scattered fragments embedded in a matrix of different agricultural uses. As a result of this agricultural expansion, habitat loss and fragmentation have become the main drivers of biodiversity loss. Understanding the long-term effects of agricultural management on populations is of great importance for the development of successful conservation strategies. Our study uses genetic data to determine the effect of agricultural management practices on the population structure of a common tropical forest rodent (*Heteromys desmarestianus goldmani*). We sampled 136 individuals from one forest fragment and three coffee farms representing varying degrees of management intensity in southern Mexico. Using microsatellite markers, we evaluated the genetic structure of *H. d. goldmani* in the study area. Our results show higher genetic differentiation and lower connectivity for individuals within high and medium intensity coffee farms than for those near and within the forest fragments. Our results suggest that the population structure observed is driven by landscape characteristics other than distance.

**Electronic supplementary material:**

The online version of this article (10.1007/s10592-017-1016-9) contains supplementary material, which is available to authorized users.

## Introduction

**Fig. 1:**
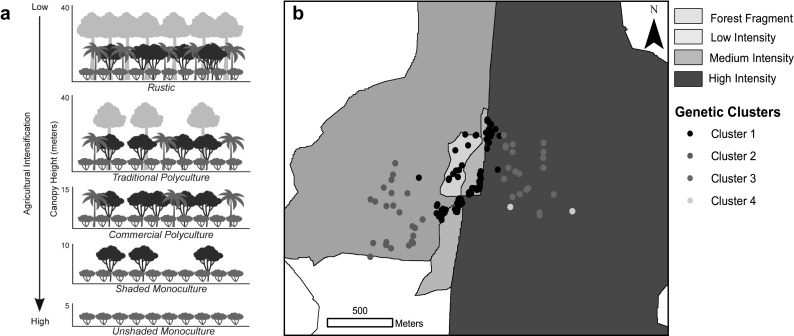
**a** Coffee production types based on Moguel and Toledo (1999). **b** Sampling sites and Geneland clustering results. Each circle represents an individual sampled, and each color represents cluster membership. (Color figure online)

Agricultural production is expanding at rapid rates in the tropics. As a consequence, most tropical forests exist as fragments embedded within a mosaic of agricultural land (Perfecto and Vandermeer 2008). Organisms that inhabit these landscapes must be able to persist within agricultural lands or navigate through them to reach habitable patches (Levins 1969). Therefore, the development of successful conservation strategies requires an understanding of the effect of agricultural production and intensification on population persistence. Agricultural intensification is the transition from traditional production systems (e.g., crop rotation, polycultures) to systems with industrial management practices (e.g., monocultures, use of agrochemicals) (Perfecto et al. 2009). For example, coffee production in Latin America falls along an intensification gradient ranging from rustic polyculture to unshaded monocultures (Moguel and Toledo 1999; Fig. 1a). This has made coffee production a model system for studies of the effects of agricultural intensification on biodiversity. Studies in coffee agroecosystems have shown a decrease in biodiversity as agricultural management intensity increases for many species, such as ants, birds, trees, bees, and bats (Perfecto and Vandermeer 2015). Less is known about the effects that management practices have on the dispersal and gene flow of species in this system.

Recently, with the development of new molecular techniques, indirect measures of dispersal can be used for studies of population connectivity (Manel et al. 2003). Gene flow can decrease with fragmentation, generating population structure within a species and increasing the impact of genetic drift (Frankel and Soulé 1981). With genetic measurements, we can evaluate the negative effects of land use change and fragmentation (e.g., loss of genetic diversity, increased differentiation) (Manel et al. 2003).

This study aims to increase our knowledge about the historical response of tropical terrestrial small mammals to agricultural intensification and forest fragmentation in a coffee agroecosystem. As common members of the animal community, small mammals play important ecological roles (Lidicker 1975), but may be negatively affected by human-driven landscape modifications (Gibson et al. 2013). *Heteromys desmarestianus goldmani*, a common rodent in southern Mexico, is known to prefer forested environments (Fleming 1983), and consuming and/or dispersing a variety of seeds in tropical forests (Martinez-Gallardo and Sanchez-Cordero 1993). This species, a yearlong breeder (Fleming 1983), has a home range of approximately 100 m^2^. This is a small home range when compared to other groups of small mammals such as *Peromyscus* spp., with home ranges averaging 2000 m^2^ (Scheibe 1984).

We studied the effect of different coffee management practices on the population genetic structure of *H. d. goldmani* with the goal of addressing the following questions. Can *H. d. goldmani* persist in a coffee agricultural matrix? If so, what is the nature of the population structure and does it vary between coffee farms and a forest fragment? Because *H. d. goldmani* is a forest specialist, we expect the species to be present in the forest fragment and within coffee farms that are either close to forest edges or within a coffee matrix of high quality (e.g., low management intensity). Additionally, we expect individuals within the forest fragment to show higher connectivity (i.e., less subpopulation genetic differentiation), than those found within the coffee farms.

## Materials and methods

This study was conducted at the Finca Irlanda Research Station located in the tropical montane region of Soconusco in Chiapas, Mexico. The area contains many coffee farms varying in management intensity, ranging from rustic to unshaded monocultures with forest patches scattered between them (Fig. 1b, see Supplementary Material). The age of the farms ranges from 60 to 100 years. Although management practices vary over time, the farms included in this study have had similar management practices for at least 17 years (Perfecto and Vandermeer 2002).

Samples were collected from four sites: one forest fragment and three coffee farms of various management levels that are adjacent to the forest fragment (Fig. 1b). Coffee farms were named after the level of management intensity (i.e., low, medium, and high) based on the Moguel and Toledo (1999) classification system. Our sampling covered an area of approximately 1.6 km^2^ (see Supplementary Material). Sex and GPS coordinates for each individual sample were recorded (Table S1, Supplementary Material), and DNA extracted from ear tissue samples. Microsatellite primers were designed specifically for *H. d. goldmani* by the Savannah River Ecology Laboratory. All samples were genotyped for 11 microsatellite loci (Table S2, Supplementary Information). We estimated relatedness (r) within and between sites to: (1) identify siblings and (2) assess bias in dispersal of males and females. We calculated three different relatedness estimators (Ritland 1996; Queller and Goodnight 1989; Lynch and Ritland (1999) using GenAlEx (Peakall and Smouse 2012).

We estimated the number of genetic units (K) and the locations of breaks in gene flow that delineate these clusters using Geneland 4.0.3 (Guillot et al. 2008), a spatial Bayesian clustering method. The analysis included 20 independent runs, using a range of K from 1 to 10. After determining the optimal number of genetic units or populations (K), a separate run was performed for the assignment of individuals (see Supplementary information for details). For the genetic clusters identified by Geneland we measured genetic diversity by quantifying observed heterozygosity (*H*_*O*_), expected heterozygosity (*H*_*E*_) and fixation index (*F*_*IS*_) GenAlEx (Peakall and Smouse 2012). Additionally, we calculated the allelic richness (AR) in ADZE (Szpiech et al. 2008) using, as our sample size, the number of individuals present in the smallest cluster. To assess differences in genetic diversity between clusters we used values for each measure (i.e., *F*_*IS*_, *H*_*E*_, *H*_*O*_, *AR*) for each locus at each cluster and conducted a bootstrapping analysis of the mean in R 3.2.4 (2016). To assess genetic differentiation between groups we calculated *F*_*ST*_ (Wright 1951) using Arlequin (Excoffier and Lischer 2010). We also measured *F*_*ST*_ for each sex separately to check for signals of sex biased dispersal.

We examined the correlation between genetic distance and geographic distance (i.e., isolation-by-distance) using Mantel tests (Mantel 1967) as implemented in GenAlEx (Peakall and Smouse 2012). Genetic distances were measured as individual pairwise *a*_*r*_ values (Rousset 2000) calculated in Genepop (Rousset 2008). Geographic distances between individuals were calculated with GPS locations using the geographical information system program ArcView version 3.1 (ESRI, California, USA).

## Results

A total of 61 alleles were scored at 11 loci in all *H. d. goldmani* samples with an average of 7.8 alleles per locus (range 3–13). Primer and locus information is in Table S2 (Supplementary Material). Results for the relatedness estimators (Table S3, Supplementary Material) indicate low relatedness of individuals within and between sampling sites. However, 0.2% of individual pairs show relatedness values of > 0.25. Although males appear to have lower levels of relatedness (Table S3, Supplementary Material), we were not able to detect significantly different patterns between males and females from each cluster (Table S6, Supplementary Material). This may be due to the low number of males present in the study, i.e. 11% of all samples (Table S3, Supplementary Material).

**Fig. 2:**
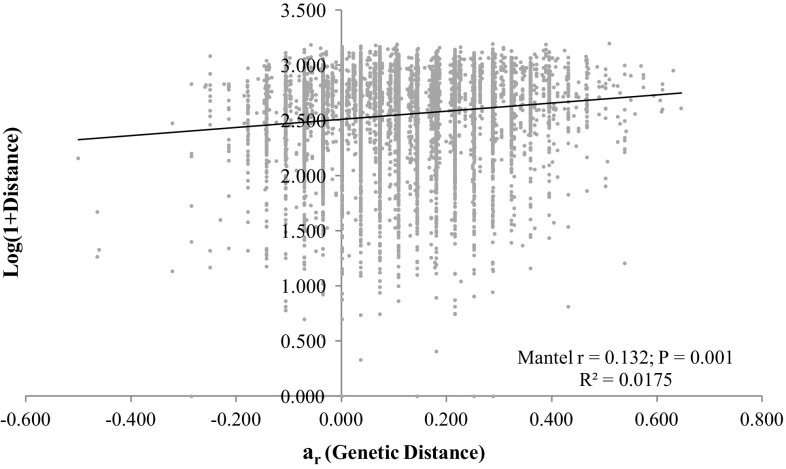
Regression showing isolation-by-distance results for all pairs of individuals sampled. Each dot represents a pair of individuals and the line represents the best fit for the data

Geneland genetic clustering analysis was done with and without closely related individuals (r > 0.25). Results did not change; thus, we present results using all individuals sampled. Genetic structure emerged from Geneland, with a K of 4 as the optimal value in all the 20 runs (Table S4). Geneland assigned most individuals to one of four clusters: (1) Individuals from the forest fragment, low intensity coffee and individuals from medium and high intensity coffee close to the forest edge (Cluster 1; Fig. 1b); (2) individuals from the medium intensity coffee farm (Cluster 2; Fig. 1b); (3) individuals from the high intensity coffee farm (Cluster 3; Fig. 1b); and (4) two individuals from the high intensity coffee farm (Cluster 4; Fig. 1b), suggesting the presence of further structuring in unsampled areas of the farm.

Interestingly, we observed that forest individuals are all assigned to a single cluster regardless of the distance between them (Fig. 1b; North to South). Samples up to 700 m from each other were assigned to the same cluster. On the other hand, we observe that this pattern does not hold for the medium and high intensity coffee farms where individuals were assigned to different clusters depending on their distance from the forest suggesting some barrier to movement (Fig. 1b; East to West). Individuals as close as 90 m from each other were assigned to different clusters. Results show no significant difference in genetic diversity measures (i.e. *H*_*E*_, *H*_*O*_, *F*_*IS*_, *AR*) among the three major clusters (Supplemental Information Table S5 and Fig. S2). The measure of genetic differentiation, *F*_*ST*_ indicated low but significant levels of differentiation between clusters (Table 1; P < 0.005).

**Table 1:** F_ST_ values for Geneland genetic clusters 1–3

	Cluster 1	Cluster 2	Cluster 3
Cluster 1	0		
Cluster 2	0.02347*	0	
Cluster 3	0.02266*	0.02035*	0

Cluster 4 was not included in this analysis due to the low samples size (n = 2). The asterisk (*) indicates values are statically significant (P < 0.5)

A significant, but weak positive relationship was found between genetic distance and geographic distance between all sample pairs (r = 0.132; P = 0.001; Fig. 2). This result suggests that isolation-by-distance (IBD) explains a small proportion of the observed genetic structure and other landscape factors might be driving the patterns observed in the cluster analyses.

## Discussion

Our results show that *H. d. goldmani* can persist within coffee farms and confirm that some population structure exists in this coffee agroecological landscape. Isolation by distance was significant but weak, suggesting that the composition of the agricultural matrix (e.g. vegetation complexity, coffee density), could limit gene flow. Resource availability in the coffee farms may explain some of the observed genetic structure. Coffee agroecosystems with low to medium management intensity (e.g., polyculture, shade grown, limited use of pesticides) have a higher vegetation complexity (Moguel and Toledo 1999) and a forest-like understory. In contrast, high intensity coffee agroecosystems present an understory substantially different from a natural forest, raising the question of how *H. d. goldmani*, a forest dwelling rodent, can persist in what must be a harsh environment for it. The mountainous landscape in which these farms occur makes some areas inaccessible to production. Farms usually have several steep ravines that are rarely planted or managed. These areas could be serving as small oases providing the resources the mice need to survive, but only in these very local micro habitats.

The literature reports only a few studies that examined the population structure of a heteromyid species (Schmidt et al. 1989; Rios et al. 2016). These studies found small genetic distances between forest populations of *Heteromys gaumeri* (Schmidt et al. 1989) using protein electrophoresis, and no genetic structure based on mitochondrial DNA for *H. nelsoni*, (Rios et al. 2016). Both species have a different ecology and distribution than *H. desmarestianus* and may not be directly comparable. Other studies examining the population structure of small mammals in agricultural landscapes have reported a variety of genetic responses to matrix composition and fragmentation (Gauffre et al. 2008; Banks et al. 2005), showing that species responses to the matrix depend on the organism’s life history. Generalist small mammal species have been reported to show high levels of connectivity between natural and agricultural lands (Gauffre et al. 2008), while specialist species show the opposite pattern of restricted dispersal (Banks et al. 2005). Our results for *H. d. goldmani* suggest that the coffee agricultural matrix may be permeable enough to facilitate dispersal and gene flow. However, the degree of permeability varies depending on other landscape characteristics, which could be linked to management practices.

Other studies across many different taxa, including small mammals, have identified natal habitat preference induction (NHPI) as a potential driver of population structure (Davis and Stamps 2004). If this is present in our system, *H. d. goldmani* individuals will prefer environments like the one they were born in. In our case, individuals born in either coffee or forest environments would preferentially seek those same conditions, thus reducing gene flow across the landscape (Davis and Stamps 2004).

Considering that a moderately intensified production system, such as the ones included in this study, appears to limit connectivity in *H. d. goldmani*, it is likely that other production practices with higher levels of intensification (e.g., soy bean or maize production) will represent strong barriers to gene flow for forest dwelling small mammals. However, more research is needed in this area to understand the management practices that drive these genetic patterns. Replicating this study in other forest fragments and coffee farms in the area would help us gain a broader understanding of the effects of land use change and fragmentation on small mammal communities at a broader scale. Moving forward, it is important also to understand patterns of connectivity, and more importantly gene flow, among populations in natural continuous landscapes. Finally, our study demonstrates the impact of the agricultural matrix in dispersal of a species and the importance of understanding long-term responses of populations to these changes.

## Electronic supplementary material

Below is the link to the electronic supplementary material.

Supplementary material 1 (DOCX 370 KB)
