# Supplementary material for: Effect of coffee agriculture management on the population structure of a forest dwelling rodent (Heteromys desmarestianus goldmani)
Source: Conserv Genet. 2017 Oct 9;19(2):495–9. doi: 10.1007/s10592-017-1016-9 (PMC5862946; doi:10.1007/s10592-017-1016-9)
Supplement: Supplementary file 1 — Supplementary material 1 (DOCX 370 KB) [file 10592_2017_1016_MOESM1_ESM.docx]

Effect of coffee agriculture management on the population structure of a forest dwelling rodent (*Heteromys desmarestianus goldmani*)

Conservation Genetics

Otero-Jiménez B, Vandermeer JH, Tucker PK

Correspondence: Beatriz Otero-Jiménez, Address: Department of Ecology and Evolutionary Biology, University of Michigan, 2019 Kraus Nat. Sci. Bldg., 830 North University, Ann Arbor, MI  48109-1048, Fax: 734 763 0544, Email: botero@umich.edu

**Supplemental Material**

**Methodology**

Study site – Our study site is dominated by coffee production. Farms included in this study are surrounded by other coffee farms.

Sampling strategy – To facilitate continuous animal collection across this complex landscape we sampled the study area by following roads and trails within the farms and the forest fragment covering an area of approximately 1.6 km^2^. Two traps were placed on each side of the trail or road every 10 meters (i.e., 4 traps at each sampling station). Traps were placed 5 and 10 meters into the coffee plantation or forest from the trail or road. To increase the area sampled, traps were set at each trap station for a single night.

Ear tissue samples were preserved in 20% DMSO buffer saturated with NaCl. Mice were captured using 22.9 x 7.6 x 8.9 cm Sherman live traps.  Sex and GPS coordinates for each individual sample were recorded. Animals were handled in accordance with the University of Michigan’s Committee on Use and Care of Animals.

DNA extraction – We obtained ear tissue samples from 136 adult and juvenile *H. d. goldmani* within our sampling area. PCR reaction mixes were made up of 1 μL 10X buffer, 1 μL dNTPs, 0.6 μL 50 mM MgCl_2_, 0.25 μL 10 μM fluorescently labeled forward primers and unlabeled reverse primers, 5.9 μL water, 0.045 μL Platinum Taq Polymerase (Invitrogen), and 1 μL DNA extract. PCRs were performed using the cycling profile: 2-min denaturation at 94 ^o^C; 40 cycles of 30s at 94 ^o^C, 30 s at varying annealing temperatures (see Table S2 Online Resources), 30 s at 72 ^o^C; and a final extension at 72 ^o^C for 10 min. Gel electrophoresis was performed on a 1% agarose gel to verify amplifications. Samples were run on Applied Biosystems DNA sequencer (Model 3730XL) at the University of Michigan DNA Sequencing Core. Allele sizes were scored using GeneMarker V 1.5 (Softgenetics). All loci were polymorphic in all sampling locations. Loci were tested for the presence of scoring errors and null alleles using Micro-Checker (Van Oosterhout et al. 2004). We tested for deviations from Hardy-Weinberg equilibrium (HWE) and for linkage disequilibrium at all loci and collecting sites (e.g., coffee farms of different intensities and forest fragment) using Arlequin 3.5.1.3 (Excoffier and Lischer 2010). Bonferroni corrections were applied to determine significance of HWE and linkage results. We estimated relatedness (r) within and between sites to check that individuals sampled were not siblings. We calculated pair-wise values of r for all individuals using GenAlEx (Peakall and Smouse 2012).

Population Structure Analyses

Geneland – The Geneland analysis included 20 independent runs with 10 000 000 MCMC iterations and 10 000 thinning (i.e., saving results from one iteration every 10 000), while varying K from 1 to 10. Correlated and null allele model options were activated and the potential error for spatial coordinates was set at 10 m. We used default settings for all other parameters. Optimal K was inferred from the run with the greatest average likelihood. After determining the optimal number of subpopulations (K), a separate run was performed for the assignment of individuals. For these runs, K was set to the previously inferred optimal number of subpopulations, the run parameters were 5 000 000 MCMC and 5 000 thinning. We calculated the posterior probability of subpopulation membership for each pixel of the spatial domain (500 x 500 pixels) with a burn-in of 100 for the run with the highest posterior probability.

**Tables and Figures**

Table S1. Sampling information for each individual. Including individual identification number, sex, sample coordinates and sampling site. For sex values (-) denotes missing information.

| **Individual ID** | **Sex** | **Latitude** | **Longitude** | **Sampling Site** |
| --- | --- | --- | --- | --- |
| 79 | F | 15.1726 | -92.33389 | C-High |
| 80 | F | 15.17276 | -92.33364 | C-High |
| 81 | F | 15.17291 | -92.33379 | C-High |
| 82 | M | 15.17295 | -92.33356 | C-High |
| 83 | F | 15.17258 | -92.33395 | C-High |
| 84 | F | 15.17314 | -92.33371 | C-High |
| 85 | F | 15.17301 | -92.33397 | C-High |
| 86 | M | 15.17294 | -92.33384 | C-High |
| 87 | F | 15.1732 | -92.33374 | C-High |
| 88 | F | 15.17354 | -92.33361 | C-High |
| 117 | F | 15.17377 | -92.33381 | C-High |
| 118 | F | 15.17377 | -92.33388 | C-High |
| 119 | F | 15.17385 | -92.33388 | C-High |
| 144 | F | 15.17324 | -92.3333 | C-High |
| 145 | F | 15.1728 | -92.33302 | C-High |
| 146 | F | 15.17285 | -92.3327 | C-High |
| 147 | F | 15.17207 | -92.33257 | C-High |
| 148 | F | 15.17216 | -92.33258 | C-High |
| 149 | F | 15.17061 | -92.33315 | C-High |
| 150 | F | 15.17072 | -92.33239 | C-High |
| 151 | F | 15.17224 | -92.33206 | C-High |
| 152 | F | 15.17048 | -92.33188 | C-High |
| 153 | F | 15.17034 | -92.3316 | C-High |
| 154 | F | 15.16818 | -92.33226 | C-High |
| 155 | F | 15.17233 | -92.32987 | C-High |
| 156 | M | 15.17001 | -92.3327 | C-High |
| 157 | F | 15.17174 | -92.33208 | C-High |
| 158 | F | 15.1693 | -92.33111 | C-High |
| 159 | F | 15.16939 | -92.33098 | C-High |
| 160 | M | 15.16902 | -92.33158 | C-High |
| 161 | F | 15.16885 | -92.3321 | C-High |
| 162 | F | 15.17072 | -92.32989 | C-High |
| 163 | F | 15.17136 | -92.3299 | C-High |
| 164 | F | 15.17175 | -92.32988 | C-High |
| 165 | F | 15.16899 | -92.32915 | C-High |
| 166 | F | 15.16791 | -92.32783 | C-High |
| 167 | F | 15.16781 | -92.33017 | C-High |
| 168 | F | 15.16765 | -92.33016 | C-High |
| 45 | F | 15.17034 | -92.33633 | C-Low |
| 46 | F | 15.17004 | -92.33662 | C-Low |
| 47 | F | 15.16996 | -92.33665 | C-Low |
| 48 | F | 15.17187 | -92.33617 | C-Low |
| 51 | F | 15.17179 | -92.33537 | C-Low |
| 52 | F | 15.17219 | -92.33525 | C-Low |
| 53 | F | 15.17219 | -92.33525 | C-Low |
| 54 | F | 15.17089 | -92.33571 | C-Low |
| 75 | F | 15.17165 | -92.33633 | C-Low |
| 76 | M | 15.17122 | -92.33612 | C-Low |
| 77 | M | 15.17146 | -92.33632 | C-Low |
| 78 | F | 15.17157 | -92.33624 | C-Low |
| 112 | F | 15.17059 | -92.3359 | C-Low |
| 113 | F | 15.17056 | -92.33612 | C-Low |
| 114 | F | 15.17015 | -92.33589 | C-Low |
| 28 | M | 15.16807 | -92.33707 | C-Medium |
| 29 | - | 15.16798 | -92.33717 | C-Medium |
| 30 | M | 15.16787 | -92.33692 | C-Medium |
| 31 | F | 15.16801 | -92.33698 | C-Medium |
| 32 | M | 15.16779 | -92.33719 | C-Medium |
| 33 | M | 15.16802 | -92.33687 | C-Medium |
| 34 | - | 15.16759 | -92.33735 | C-Medium |
| 35 | F | 15.16735 | -92.33716 | C-Medium |
| 89 | F | 15.16779 | -92.33752 | C-Medium |
| 90 | F | 15.16837 | -92.33746 | C-Medium |
| 91 | F | 15.16828 | -92.33751 | C-Medium |
| 115 | F | 15.17286 | -92.33467 | C-Medium |
| 116 | F | 15.17283 | -92.33472 | C-Medium |
| 120 | F | 15.16685 | -92.33906 | C-Medium |
| 121 | F | 15.16635 | -92.33918 | C-Medium |
| 122 | - | 15.16739 | -92.33852 | C-Medium |
| 123 | F | 15.16587 | -92.33921 | C-Medium |
| 124 | M | 15.16597 | -92.33929 | C-Medium |
| 125 | F | 15.16572 | -92.3395 | C-Medium |
| 126 | F | 15.16499 | -92.34225 | C-Medium |
| 127 | F | 15.16575 | -92.34114 | C-Medium |
| 128 | F | 15.16593 | -92.3411 | C-Medium |
| 129 | F | 15.16553 | -92.34077 | C-Medium |
| 130 | F | 15.16695 | -92.33924 | C-Medium |
| 131 | F | 15.16785 | -92.34017 | C-Medium |
| 132 | F | 15.16831 | -92.34061 | C-Medium |
| 133 | F | 15.16911 | -92.34074 | C-Medium |
| 134 | F | 15.16827 | -92.3411 | C-Medium |
| 135 | F | 15.16857 | -92.3419 | C-Medium |
| 136 | F | 15.1687 | -92.34223 | C-Medium |
| 137 | F | 15.17104 | -92.34046 | C-Medium |
| 138 | F | 15.17031 | -92.34061 | C-Medium |
| 139 | F | 15.16998 | -92.34073 | C-Medium |
| 140 | F | 15.16946 | -92.34129 | C-Medium |
| 141 | F | 15.17011 | -92.33878 | C-Medium |
| 142 | F | 15.16909 | -92.3396 | C-Medium |
| 143 | M | 15.16588 | -92.34206 | C-Medium |
| 1 | F | 15.16832 | -92.33596 | Forest Fragment |
| 2 | - | 15.16832 | -92.33596 | Forest Fragment |
| 3 | - | 15.16835 | -92.33582 | Forest Fragment |
| 4 | F | 15.16866 | -92.33596 | Forest Fragment |
| 5 | F | 15.16869 | -92.33577 | Forest Fragment |
| 6 | M | 15.1687 | -92.33577 | Forest Fragment |
| 7 | F | 15.16839 | -92.33581 | Forest Fragment |
| 8 | F | 15.1684 | -92.33577 | Forest Fragment |
| 9 | F | 15.16845 | -92.33605 | Forest Fragment |
| 10 | M | 15.16806 | -92.33602 | Forest Fragment |
| 11 | F | 15.16835 | -92.33593 | Forest Fragment |
| 12 | F | 15.16875 | -92.33589 | Forest Fragment |
| 13 | F | 15.16868 | -92.33595 | Forest Fragment |
| 14 | F | 15.16846 | -92.33567 | Forest Fragment |
| 15 | F | 15.16846 | -92.33567 | Forest Fragment |
| 16 | F | 15.16805 | -92.33621 | Forest Fragment |
| 17 | F | 15.16787 | -92.33605 | Forest Fragment |
| 18 | F | 15.16807 | -92.33601 | Forest Fragment |
| 19 | F | 15.16798 | -92.33611 | Forest Fragment |
| 20 | F | 15.16873 | -92.33579 | Forest Fragment |
| 21 | F | 15.16789 | -92.33613 | Forest Fragment |
| 22 | F | 15.16912 | -92.33522 | Forest Fragment |
| 23 | F | 15.16915 | -92.3352 | Forest Fragment |
| 24 | F | 15.16802 | -92.33533 | Forest Fragment |
| 25 | F | 15.169 | -92.3352 | Forest Fragment |
| 26 | F | 15.16909 | -92.33504 | Forest Fragment |
| 27 | F | 15.16909 | -92.33504 | Forest Fragment |
| 49 | F | 15.17197 | -92.33389 | Forest Fragment |
| 50 | F | 15.17236 | -92.33376 | Forest Fragment |
| 55 | F | 15.16972 | -92.33443 | Forest Fragment |
| 56 | F | 15.1696 | -92.33442 | Forest Fragment |
| 57 | F | 15.16932 | -92.33466 | Forest Fragment |
| 58 | F | 15.16945 | -92.33443 | Forest Fragment |
| 59 | F | 15.16945 | -92.33443 | Forest Fragment |
| 60 | F | 15.16932 | -92.3346 | Forest Fragment |
| 61 | F | 15.16955 | -92.33447 | Forest Fragment |
| 62 | M | 15.16936 | -92.33454 | Forest Fragment |
| 63 | F | 15.16941 | -92.33477 | Forest Fragment |
| 64 | F | 15.16943 | -92.33498 | Forest Fragment |
| 65 | F | 15.16935 | -92.33448 | Forest Fragment |
| 66 | F | 15.16985 | -92.33447 | Forest Fragment |
| 67 | F | 15.16989 | -92.33442 | Forest Fragment |
| 68 | F | 15.16984 | -92.33442 | Forest Fragment |
| 69 | F | 15.17002 | -92.33436 | Forest Fragment |
| 70 | F | 15.16985 | -92.33447 | Forest Fragment |
| 71 | F | 15.1705 | -92.33437 | Forest Fragment |
| 72 | F | 15.17019 | -92.33439 | Forest Fragment |
| 73 | F | 15.17029 | -92.33443 | Forest Fragment |
| 74 | F | 15.17029 | -92.33443 | Forest Fragment |

| **Locus** | **Forward** | **Annealing Temp (°C)** | **Repeat** | **A_R_** | **H_O_** | **H_E_** | **F_IS_** |
| --- | --- | --- | --- | --- | --- | --- | --- |
| HET-1 | **F-** CATTTGCCTTTCTCAGTGGC | 68 | AC | 10.50 | 0.669 (0.028) | 0.772 (0.019) | 0.131 (0.044) |
|  | **R-** GTGGGTCGAGATTTCACTGG |  |  |  |  |  |  |
| HET-4 | **F-** TCATGCAGGGAAGTCTGGG | 65 | AC | 6.25 | 0.631 (0.097) | 0.750 (0.005) | 0.159 (0.130) |
|  | **R-** TAGGATGATACTCATTCCCAAACAAGC |  |  |  |  |  |  |
| HET-23 | **F-** TCATATCTGGCAGCAAGAAGC | 68 | ATC | 6.00 | 0.646 (0.025) | 0.665 (0.023) | 0.026 (0.048) |
|  | **R-** GACAAGCATATTTGGTTGATTGC |  |  |  |  |  |  |
| HET-27 | **F-** TGCTCTCTTTCTCACACTGGC | 68 | TGC | 7.50 | 0.643 (0.024) | 0.690 (0.017) | 0.068 (0.030) |
|  | **R-** GGCAACTATGGTGATCCTGG |  |  |  |  |  |  |
| HET-32 | **F-** AGGGTCTGGACTAGGCATGG | 61 | ACC | 4.50 | 0.665 (0.040) | 0.691 (0.023) | 0.038 (0.048) |
|  | **R-** GTCGGCTCAGAGATGCTCC |  |  |  |  |  |  |
| HET-34 | **F-** CCATTCACTCATCTGTTAATCATAAGG | 68 | AGT | 9.00 | 0.761 (0.035) | 0.845 (0.011) | 0.100 (0.036) |
|  | **R-** CTGGGTAGATGGCTCTTGCC |  |  |  |  |  |  |
| HET-37 | **F-** ACTGCAATGTCCGGTTTGC | 58 | ATT | 7.00 | 0.791 (0.016) | 0.746 (0.008) | -0.060 (0.017) |
|  | **R-** TCAACAAGACCCACACAGGC |  |  |  |  |  |  |
| HET-41 | **F-** CCTGCCTACCTCTCTCATCC | 62 | ATC | 4.25 | 0.330 (0.038) | 0.615 (0.009) | 0.464 (0.060) |
|  | **R-** GGCATTCTACCACAGAGTAGCC |  |  |  |  |  |  |
| HET-42 | **F-** GACTATAGGCATCACTATGTCCAGC | 62 | ATT | 5.50 | 0.755 (0.034) | 0.719 (0.015) | -0.049 (0.028) |
|  | **R-** GAATGCAATACCCATGTCCC |  |  |  |  |  |  |
| HET-46 | **F-** CACAATTCCCAATGTGGTGG | 62 | CGG | 9.25 | 0.673 (0.117) | 0.841 (0.019) | 0.208 (0.122) |
|  | **R-** CATTGACCAGAATCAAGAAATTAGG |  |  |  |  |  |  |
| HET-56 | **F-** GCTCTGTGAGTCAGTAGTTGAGGG | 64 | AAAC | 2.25 | 0.375 (0.055) | 0.354 (0.014) | -0.052 (0.120) |
|  | **R-** CTGTGCCTCATGCTTTCTCC |  |  |  |  |  |  |
| HET-57 | **F-** TGTATGTGTTCTCTATGAATCTCTAACCC | 62 | AAAG | 9.00 | 0.793 (0.045) | 0.816 (0.014) | 0.028 (0.05) |
|  | **R-** CCAACACAAGTGTATACTGATATTATTTCC |  |  |  |  |  |  |

Table S2. Forward and reverse sequences, annealing temperatures, repeat sequence and the means for: allelic richness (AR), observed heterozygosity (H_O_), expected heterozygosity (H_E_) and fixation index (FIS) for each loci.

Table S3. Mean for each relatedness estimator; Ritland 1996 (RI), Queller and Goodnight 1989 (QGM) and Lynch and Ritland 1999 (LRM). Results for within and between sampling sites followed by results by sex.

| **Sampling Site** | **Relatedness estimate** | | | **No. samples** |
| --- | --- | --- | --- | --- |
|  | **RI** | **QGM** | **LRM** |  |
| Forest | -0.011 | -0.021 | -0.010 | 49 |
| C-Low | -0.037 | -0.071 | -0.036 | 15 |
| C-Medium | -0.016 | -0.029 | -0.014 | 36 |
| C-High | -0.016 | -0.030 | -0.016 | 35 |
| Forest + C-High | -0.007 | -0.012 | -0.006 | 84 |
| Forest + C-Medium | -0.006 | -0.012 | -0.006 | 85 |
| Forest + C-Low | -0.008 | -0.016 | -0.008 | 64 |
| C-High +C-Medium | -0.008 | -0.014 | -0.007 | 71 |
| C-High + C-Low | -0.011 | -0.020 | -0.010 | 50 |
| C-Medium + C-Low | -0.011 | -0.020 | -0.010 | 51 |
| Males | -0.037 | -0.072 | -0.036 | 15 |
| Females | -0.005 | -0.008 | -0.004 | 117 |
| All | -0.004 | -0.007 | -0.004 | 135 |

Table S4. Results for individual-based Geneland clustering analyses for all sampling sites. The 20 runs were ranked by decreasing value of likelihood and the estimated K value is presented for each run.

| **Geneland** |  |
| --- | --- |
| Likelihood | K |
| -2303 | 4 |
| -2318 | 4 |
| -2326 | 4 |
| -2328 | 4 |
| -2330 | 4 |
| -2331 | 4 |
| -2337 | 4 |
| 2339 | 4 |
| -2352 | 4 |
| -2354 | 4 |
| -2357 | 4 |
| -2364 | 4 |
| -2365 | 4 |
| -2365 | 4 |
| -2367 | 4 |
| -2368 | 4 |
| -2369 | 4 |
| -2377 | 4 |
| -2379 | 4 |
| -2389 | 4 |

Table S5. Genetic diversity measures for each genetic cluster identified by Geneland including values for the mean and standard error (SE). Cluster 4 was not included in this analysis due to the low sample size (n=2).

| **Cluster** | **N** | **A_R_** | **H_O_** | **H_E_** | **F_IS_** |
| --- | --- | --- | --- | --- | --- |
| Cluster 1 | 93 | 4.89 (0.42) | 0.641 (0.052) | 0.698 (0.040) | 0.084 (0.050) |
| Cluster 2 | 22 | 5.13 (0.57) | 0.607 (0.069) | 0.699 (0.041) | 0.127 (0.083) |
| Cluster 3 | 18 | 5.12 (0.54) | 0.671 (0.060) | 0.690 (0.046) | 0.037 (0.042) |

Table S6. F_ST_ values for Geneland clusters by sex. The asterisk (*) indicates values are statistically significant (P<0.5).

Females

|  | **Cluster 1** | **Cluster 2** | **Cluster 3** | **Cluster 4** |
| --- | --- | --- | --- | --- |
| **Cluster 1** | 0 |  |  |  |
| **Cluster 2** | 0.02396* | 0 |  |  |
| **Cluster 3** | 0.02458* | 0.02524* | 0 |  |
| **Cluster 4** | -0.03646 | 0.02524 | 0.01518 | 0 |

Males

|  | **Cluster 1** | **Cluster 2** | | | **Cluster 3** | |
| --- | --- | --- | --- | --- | --- | --- |
| **Cluster 1** | 0 |  |  | | | |
| **Cluster 2** | 0.08956 | 0 |  | | | |
| **Cluster 3** | 0.03176 | 0.04132 | | 0 | |  |

Figure S1. Distribution of relatedness estimator (LRM) within each sampling site: (a) Forest Fragment, (b) High Intensity Coffee, (c) Medium Intensity Coffee, and (d) Low Intensity Coffee.

a. b.

c. d.

Figure S2. Results showing the mean and 95% confidence interval of bootstrapping of the mean for measures of genetic diversity (e.g., allelic richness (AR), expected heterozygosity (H_E_), observed heterozygosity (H_O_) and fixation index (F_IS_)).


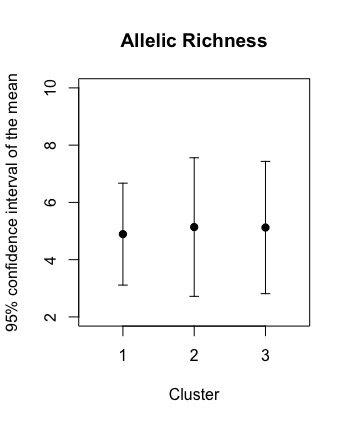


Figure S3. Relationship between relatedness estimate (LRM) and geographic distance for (a) females and (b) males.

a.

b.
